# Leptin and Immunological Profile in Obesity and Its Associated Diseases in Dogs

**DOI:** 10.3390/ijms20102392

**Published:** 2019-05-14

**Authors:** Laura Cortese, Giuseppe Terrazzano, Alessandra Pelagalli

**Affiliations:** 1 Department of Veterinary Medicine and Animal Productions, University of Naples Federico II, 80137 Naples, Italy; 2 Department of Science, University of Basilicata, 85100 Potenza, Italy; 3 Department of Translational Medical Sciences, University of Naples Federico II, 80131 Naples, Italy; 4 Department of Advanced Biomedical Sciences, University of Naples Federico II, 80131 Naples, Italy; 5 Institute of Biostructures and Bioimages (IBB), National Research Council (CNR), 80131 Naples, Italy

**Keywords:** leptin, dog, obesity, immune function, physiology, human

## Abstract

Growing scientific evidence has unveiled increased incidences of obesity in domestic animals and its influence on a plethora of associated disorders. Leptin, an adipokine regulating body fat mass, represents a key molecule in obesity, able to modulate immune responses and foster chronic inflammatory response in peripheral tissues. High levels of cytokines and inflammatory markers suggest an association between inflammatory state and obesity in dogs, highlighting the parallelism with humans. Canine obesity is a relevant disease always accompanied with several health conditions such as inflammation, immune-dysregulation, insulin resistance, pancreatitis, orthopaedic disorders, cardiovascular disease, and neoplasia. However, leptin involvement in many disease processes in veterinary medicine is poorly understood. Moreover, hyperleptinemia as well as leptin resistance occur with cardiac dysfunction as a consequence of altered cardiac mitochondrial metabolism in obese dogs. Similarly, leptin dysregulation seems to be involved in the pancreatitis pathophysiology. This review aims to examine literature concerning leptin and immunological status in obese dogs, in particular for the aspects related to obesity-associated diseases.

## 1. Introduction

Obesity is a metabolic disorder arising due to abnormal and frequently ectopic white adipose tissue deposition, as a consequence of an altered balance between energy intake and consumption, usually dependent on the body’s inefficiency to convert caloric intake in energy. Literature has been focused on the role of leptin in determining obesity and related diseases in humans. The economic impact of obesity on the world health care system stimulated research interest [1] on the underlying molecular and biochemical mechanisms of obesity-associated comorbidities such as diabetes mellitus, metabolic disorders [2], orthopaedic disease [3], respiratory dysfunction [4], and altered renal function [5]. Growing incidences of obesity highlighted its relevance in domestic animals and, in particular, in dogs [6].

From its first characterization in 1994 to today, the relationship between leptin and its involvement in supporting inflammatory processes becomes increasingly relevant also in the determinism of autoimmunity [7,8].

In this regard, white adipose tissue has been considered not only a fat storage compartment able to regulate energy homeostasis, but also a key provider of several biomolecules able to deeply modulate tissue physiology in individuals [9,10,11,12]. Leptin production is mainly based on adipocytes secretion, the level of such a hormone correlates with white adipose tissue mass and contributes to an inflammatory state in overweight and obese humans [12]. In addition to the control of the biological processes involved in energy homeostasis, leptin has systemic effects that include the regulation of endocrine function, the immune response, and haematopoiesis [13,14,15]. Therefore, obesity can be considered as a chronic and complex pathological state associated with multiple systemic and tissue-specific alterations. This review intends to examine the physiological role of leptin, the possible relationship between leptin, immune function, and metabolism in dogs, and to highlight possible perspectives for human studies.

## 2. Physiological Role of Leptin and Its Relationship with Obesity

Leptin is a hormone secreted by white adipocytes [16,17]. Through the blood-brain barrier, such a hormone reaches the hypothalamus to decrease food intake and to increase metabolism [16]. Leptin receptors, encoded by the *LEPR* gene [18,19], are expressed by hypothalamic satiety centres and are widely disseminated throughout the body—this occurrence reflects the pleiotropic nature of leptin that is involved in the control of many physiologic processes [20]. Ob-Rb, the ‘long’ isoform of the receptor, is predominantly expressed in the hypothalamus [21,22,23], while the short isoforms (Ob-Ra, Ob-Rc, Ob-Rd, and Ob-Rf) are expressed in the peripheral tissues [24,25]. Leptin receptor (*LEPR*) needs the activation of receptor associated kinases of Janus family (JAKs), which in turn induce downstream signalling involving different members of signal transducers and activators of transcription (STAT) family [26]. Leptin receptors activate a complex neural circuit involving anorexigenic (i.e., appetite-diminishing) and orexigenic (i.e., appetite-stimulating) neuropeptides to control food intake.

Moreover, leptin also stimulates the sympathetic nervous system inducing an increase in plasma norepinephrine and epinephrine concentrations via the ventromedial hypothalamus [27].

In addition to its pivotal role in the regulation of energy metabolism [28], leptin possesses other important physiological activities as the control of neuroendocrine and immune functions, and haematopoiesis [29,30]. The strict association between obesity and hematopoietic disruption evidenced the role of leptin on bone organization. The direct role for leptin in haematopoiesis has been demonstrated by the presence of Ob-R on bone marrow CD34^+^ cells as well as on lympho-haematopoietic and megakaryocytic cell lines [31,32]. Recently, Claycombe et al. [33] demonstrated that myelopoiesis recover after treatment with leptin in obese mice (*ob/ob*). Aberrant leptin levels in patients with haematological malignancies have been described, suggesting that leptin signalling is involved in the progression of haematological malignancies and could represent a useful prognostic value [34].

Relationship between leptin and obesity could be considered as a part of metabolic syndrome (MS), the pathological condition comprising of also dyslipidaemia, hyperglycaemia, and high blood pressure. It is noteworthy that obesity is related to the leptin receptor resistance mechanisms [35], including several aspects such as: (i) Interruption of leptin signalling in hypothalamic and other central nervous system (CNS) neurons; (ii) alteration of leptin transport across blood-brain barrier; (iii) hypothalamic inflammation, autophagy, and endoplasmic reticulum stress [36,37]. The development of leptin resistance and of hyperleptinemia have been widely demonstrated in humans and in domestic animals [38].

In the course of obesity and hyperleptinemia condition, an accumulation of epicardial adipose tissue has been demonstrated [39], suggesting its involvement in cardiovascular system damage. Chronic inflammation and the accumulation of epicardial fat is strongly concomitant with coronary artery disease, independent of visceral adiposity [39]. Furthermore, high circulating levels of leptin appeared to induce significant impairment of the haemostatic balance in cardiovascular diseases [40].

Moreover, leptin has been associated to hypertension and congestive heart failure (HF) in humans, dogs, and cats [38,41]. In addition, leptin accelerates atherosclerosis spreading [42].

The role of leptin and adipokines on the cardiovascular system have been largely described to be dependent on two mechanisms involving the heart or the central nervous system [43,44,45]. Leptin acts by stimulating the migration and proliferation of vascular smooth muscle cells (VSMCs) [46]. Such hormones block the vasoconstrictor action of angiotensin II and inhibits the angiotensin II-induced increase in intracellular Ca^2+^ in VSMCs through Ob-Rb [47]. Leptin shows angiogenetic effects dependent on both proliferation and migration of vascular smooth muscle cells by promoting the upregulation of vascular endothelial growth factor (VEGF) expression [48] and the cytoskeleton reorganization [49].

Acute pancreatitis is associated with high levels of leptin in serum and pancreas [50,51], suggesting the role for such a hormone as a marker for adipose tissue necrosis [52]. Intriguingly, the pancreas could secrete leptin and its protective role in pancreatitis has been described [53,54]. In agreement with this hypothesis, beneficial effects of leptin on acute pancreatitis have been evidenced in ischemia/reperfusion [54,55].

## 3. Role of Leptin in the Relationship between Obesity and Immune-Modulation.

An interesting scenario on obesity is that immune response greedily needs “energy” to be implemented. In a pathophysiological perspective, this energy can be in excess or in deficit. In this regard, food opulence is frequently associated with autoimmune diseases [7,56,57], while hyponutrition induces susceptibility to infectious diseases [58,59,60,61]. Therefore, an excess of nutrients could drive the immune system towards self-reactivity, while a defect can determine insufficient anti-infectious immune responses. In this regard, the relationship between obesity and immune modulation appears of great relevance in both human and veterinary medicine [7,56,57,62,63,64,65,66,67]. 

**Figure 1:**
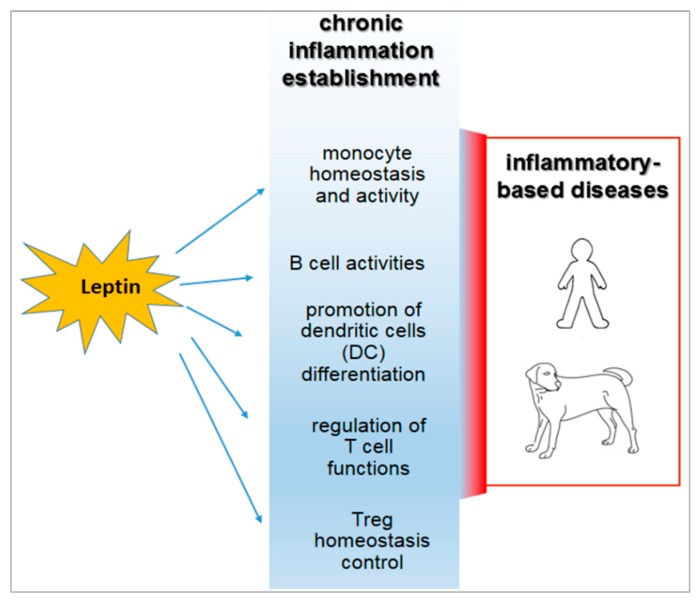
Inflammatory roles of leptin in the course of obesity and their relevance in both human and veterinary medicine.

In human and animal obesity, the secretion of leptin and other hormones from the adipose tissue appears to determine the dysregulation of the immune response [7,68,69] (Figure 1).

Moreover, leptin and its receptors are integral components of a complex physiological system evolved to regulate fuel stores and energy balance at an optimum level in mammals [70].

**Figure 2:**
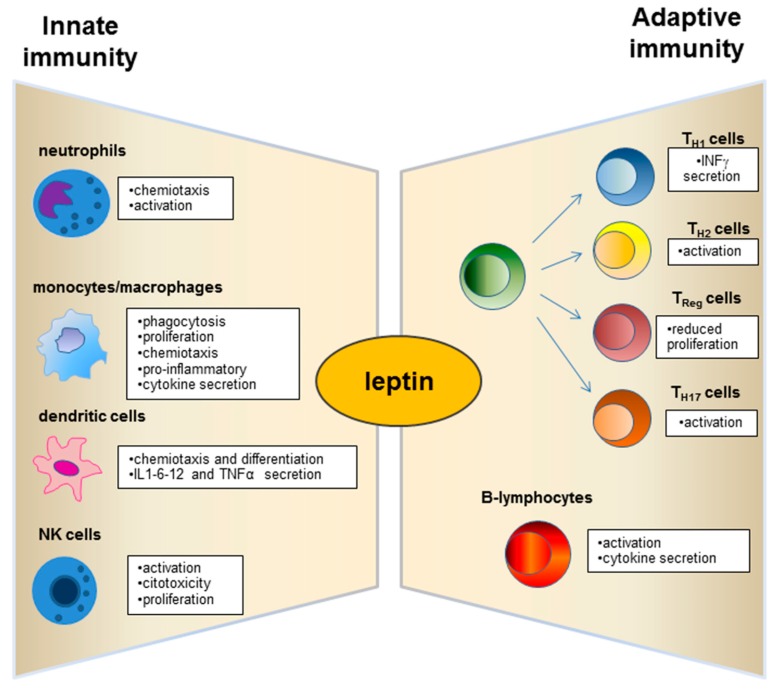
Physiological role of leptin on innate and adaptive immunity.

Leptin has structural similarities with the alpha-helix family of cytokines and its receptor (ObR) belongs to the superfamily of class I cytokine receptors [71]. Leptin receptors are expressed by immune system cells [72,73,74], and leptin possesses modulatory effects on both innate and adaptive immunity [75,76] (Figure 2). Such a hormone is currently considered a pro-inflammatory adipokine [7,8,12]. In this regard, leptin acts as an acute phase inflammatory cytokine like interleukin (IL)-1, IL-6, and tumour necrosis factor (TNF)-α [29] and is necessary for phagocytosis of bacteria by polymorph nuclear cells [77].

Several studies evidenced the involvement of leptin in activation of macrophages [78] and in their recruitment in adipose tissue [79] (Figure 2). Leptin fosters pro-inflammatory activity by monocyte [80] and promotes their production of reactive oxygen species (ROS) [81,82] (Figure 2). Furthermore, such a hormone plays an anti-apoptotic role in serum-deprived monocytes demonstrating that this adipokine could act as a growth factor for these cells [83]. Leptin also exerts chemotactic activity on neutrophils [84] and promotes their production of intracellular hydrogen peroxide [85] (Figure 2).

Neutrophils express the short form of the leptin receptor [86] that can stimulate the expression of CD11b and prevent apoptosis.

Dendritic cells (DC), a specialized cell population for antigen uptake in body tissues, express leptin receptors (Ob-R) on their surface [87]. Leptin acts on these cells, favouring their differentiation, maturation, recruitment, and survival [87,88] and modulating the signalling pathways involved in these biological processes as observed in *db/db* mice lacking leptin receptors (Ob-R) [88]. Furthermore, an important role of leptin is exercised by the activation and recruitment of the DC (Figure 2).

Deficits of leptin receptors in Natural Killer (NK) cells correlate with decreased NK number and functions [89,90].

Moreover, *LEPR*-deficient (*db/db*) mice evidenced a decrease of NK function [91].

The role of leptin in adaptive immunity has been largely demonstrated from early studies on *db/db* mice that showed high level of thymocyte apoptosis [92].

A great research interest has moved to explore the leptin role on the T and B cell population (Figure 2). Leptin acts with several mechanisms on T lymphocytes and induces the expression of the long isoform of *LEPR* in CD4^+^ T cells [93]. Such adipokine promotes activation and proliferation of T lymphocytes and enhances their cytokine production [94,95]. In addition, the leptin supplementation to a mixed lymphocyte reaction has been observed to induce a proliferation of CD4^+^ T cells [95].

Leptin regulates the adaptive immunity, also influencing activities of T Helper (Th) 1 and 2 lymphocytes [7,8,58,96]. In particular, the hormone stimulates the Th1 production of cytokines such as IL-2, interferon (IFN)-γ, TNF-α, and IL-18, and drives the differentiation of the Th17 cells mainly involved in chronic inflammation establishment [97,98].

In addition, leptin influences B-cell activities, regulating and promoting cell cycle by Bcl-2 and cyclin D activation [99].

It is of note that leptin acts on the homeostasis of a specific CD4^+^CD25^high^Foxp3^+^ T immune regulatory cell population, usually referred to as Treg [7,100,101,102,103,104]. Such cells avoid the auto reactivity of the immune system against the “self” molecular components that belong to the individual itself [7,100,101,102]. Human Treg cells display heterogeneous gene expression, phenotype, and suppressive functions [105]. This occurrence strongly correlates with the different splicing variants of the transcriptional factor FoxP3 [106]—the full-length isoform (FoxP3fl), which contains the sequences involved in the interaction with retinoic acid-related orphan receptors α and γt (RORα and RORγt), is associated with Treg function in humans [107]. In contrast, the expression of the isoform lacking exon 2 (FoxP3Δ2) correlates with dysfunction of Treg cells, since it appears to be unable to interact with RORα and RORγt [108]. FoxP3Δ2 expression has been correlated with multiple sclerosis in humans [109]. Expression of the different FOXP3 isoforms is conditioned by metabolic aspects [110] and by the exposure of Treg to the pro-inflammatory micro-environment [111]. No data over this potential functional dichotomy are available from canine or feline models.

Nutrient availability is essential for the maintenance of tissue homeostasis. In this context, the intracellular “sensor” of nutrients [112] is represented by the mammalian target of rapamycin, the mTOR molecule [113]. This serine–threonine kinase “senses” the extracellular bioavailability of amino acids, glucose, growth factors, and hormones [101,112,113,114], promotes cell metabolism and growth when the conditions are favourable; or catabolic processes when conditions are not favourable. In this context, mTOR is strongly correlated with Treg homeostasis and functions [115]. High levels of leptin correlate with a reduced number and with decreased functions of Treg cells in human autoimmune diseases [7,56]. The relationship between metabolism and cell plasticity is of great relevance, particularly for the homeostasis of immune system cells that are highly “sensitive” to bioavailable nutrients [116,117,118,119]. In this context, T effectors and Treg cells [114,120] are significantly influenced by metabolism—such an occurrence may explain why caloric excess correlates with autoimmune diseases [7,57], while hyponutrition increases susceptibility to infections [58,59,60,61]. In this negatively virtuous interplay, the high levels of leptin secreted by the adipose tissue are able to dysregulate the Treg cells and determine an increased risk of developing autoimmune diseases in obese patients [121,122,123].

Reduced Treg cells have been observed in visceral adipose tissue of obese mice and humans. However, it is unknown whether human obesity affects circulating Treg cells and whether Treg number is associated with markers of systemic inflammation or glucose intolerance. The effect of human obesity to reduce Treg levels has been addressed [124,125]. Circulating Treg cells are inversely correlated with body weight and plasma leptin levels [125].

Reduction of circulating Treg cells in obesity may be caused by their recruitment into an active inflammation site. In this regard, an upregulated expression of homing receptors—including the chemokine receptors CXCR3 and CCR7—on the surface of Treg cells and an increased accumulation of Treg cells in the spleen of obese mice have been reported [126]. Increased serum levels of adipose tissue-derived cytokines may impair Treg cell maturation and/or survival in obesity. Interestingly, the receptor of the adipokine leptin is expressed on T lymphocytes [95], including Treg cells, and leptin neutralization promotes their proliferative capacity [101]. Moreover, hypercholesteraemic LDL-R mice with defective leptin signalling exhibited improved Treg cell functions [127].

Adiposity has been associated with increased concentrations of leptin and other proinflammatory adipokines, cytokines, and acute-phase proteins [128]. The role of adiponectin in dogs still appears controversial and few data are available in the veterinary literature on the possible impact of obesity on the immune response. The effects of weight loss on canine adipokines and cytokines have been reported [2,3,129,130,131]. Several studies showed that plasma leptin concentrations correlate with body fat content in experimentally induced obese beagles [132,133]. In this regard, Sagawa et al. highlighted that the positive relationship between plasma leptin concentration and body fat content in dogs is similar to correlations reported for humans and rodents [132]. Ishioka et al. [134] showed that plasma leptin represents an index of adiposity in dogs regardless of their age, gender, and breed variations. It is well known that plasma leptin concentrations increase with weight gain and decrease with weight loss in dogs. In this regard, Jeusette et al. [135] described a decrease in ghrelin and an increase in leptin and insulin concentrations in obese beagle dogs. The same authors [135] suggested that ghrelin and leptin could play a role in dogs in the adaptation to a positive or negative energy balance, as observed in humans. Proinflammatory state directly influences glucose metabolism, resulting in decreased insulin sensitivity [128]. In fact, high-plasma leptin concentrations have been correlated to insulin resistance in humans [136] and in insulin-resistant dogs [135]. Serum leptin concentrations correlated with percentage of body fat and decreased with weight loss, whereas the involvement of other inflammatory markers in canine obesity and weight loss is still less understood. Induction of canine obesity has been shown to increase concentrations of TNF-α [137] which decreases after a weight loss program in obese dogs [2]. However, acute phase proteins appeared to be unaltered after the weight loss program [129], while the production of C-reactive protein decreased in obese dogs [2,129,130,131,138].

Van de Velde et al. [139] investigated the effect of a short-term increase in body weight on immunological variables in adult healthy beagle dogs in which weight gain and increased body condition score (BCS) were accompanied by a significantly higher leptin concentration. Subsequently, the same authors [140] described that T-cell proliferation is affected after weight gain in Beagle dogs.

Recently, concentrations of IL-6 and monocyte chemoattractant protein 1, but not IL-8, were found to be increased in overweight dogs [141], whereas other authors described decreasing concentrations of IL-8 and other interleukins with weight loss in dogs [131]. Piantedosi et al. [142] revealed no significant differences in serum TNF-α and IL-6 concentrations between obese and normal weight dogs.

Several studies have reported that the systemic circulating leptin deficiency in malnutrition is also correlated to infectious diseases [61,77,81,143,144,145] including leishmaniasis [146,147] due to defective cytokine production [84,148]. Leptin can augment host protective immune response during experimental visceral leishmaniasis (VL) [146,147]. Indeed, leptin induces the phagocytic activity of human macrophages against *L. donovani* infection by enhancing the phagolysosome formation and oxidative killing of the parasite via intracellular reactive oxygen species (ROS) generation [147].

Palatucci et al. [65] reported that obese Labrador Retrievers are characterized by the inverse correlation between leptin serum concentration and circulating Treg levels. Moreover, an increased number of cytotoxic T cell effectors and a higher IFN-γ production by cytotoxic T lymphocytes have been observed in obese dogs [65]. However, the relationship between obesity, leptin, and circulating Treg level, as well as the occurrence of systemic inflammation in dogs and in other domestic mammalians are still poorly understood.

Increased inflammatory response has been correlated with clinical exacerbation, and the immunotherapeutic role of Tregs appears to be relevant in leishmaniosis [149].

Tregs function, macrophage activation, and the proinflammatory state appear to be involved in the pathogenesis of canine leishmaniasis. Naturally *L. infantum* infected dogs expressed alteration in leptin gene transcription and low levels of circulating Treg [150]. In the same model, ineffective immune response to parasites appeared to be associated with high Treg levels [151]. Di Loria et al. [152] showed an increase in leptin mRNA expression in dogs naturally infected by *L. infantum*.

## 4. Leptin and Associated Diseases in Humans and Dogs

High body mass index represents a risk factor in both human and canine mammary inflammatory carcinomas [153,154,155,156]. How obesity can influence the development and prognosis of human breast cancer remains unknown, although several factors secreted by adipocytes including aromatase, leptin, adiponectin, oestrogens, and insulin-like growth factor-1 have been implicated [157]. Leptin may promote carcinogenesis of the mammary tissue through its interaction with the leptin receptor Ob-R [158,159,160,161,162]. Such a hormone could affect breast cancer by stimulating growth of normal mammary epithelial cells and tumour cells, tumour invasion, angiogenesis, and aromatase activity [157,162]. Obesity is considered a pro-inflammatory state and is associated with increasing circulating levels of TNF-α and IL-6 [163]. Chronic inflammation promotes tumour development [164], macrophage recruitment in mammary gland in human and murine obese subjects [165,166] and metastasis of breast tumours [167]. Notably, obesity-related macrophage infiltration of murine mammary gland reversed with caloric restriction [168].

Obesity affects progression, and metastasis in canine mammary carcinoma (MC) by recruitment of macrophages [156] (Figure 2). In this regard, macrophage infiltration of tumour areas appears to be higher in overweight or obese subjects than in lean subjects. In addition, decreased adiponectin expression and increased macrophage numbers in overweight or obese subjects associate with poor prognosis, high histological grade, and lymphatic invasion [156]. Leptin and Ob-R expression correlates with oestrogen receptor status MC [156].

**Figure 3:**
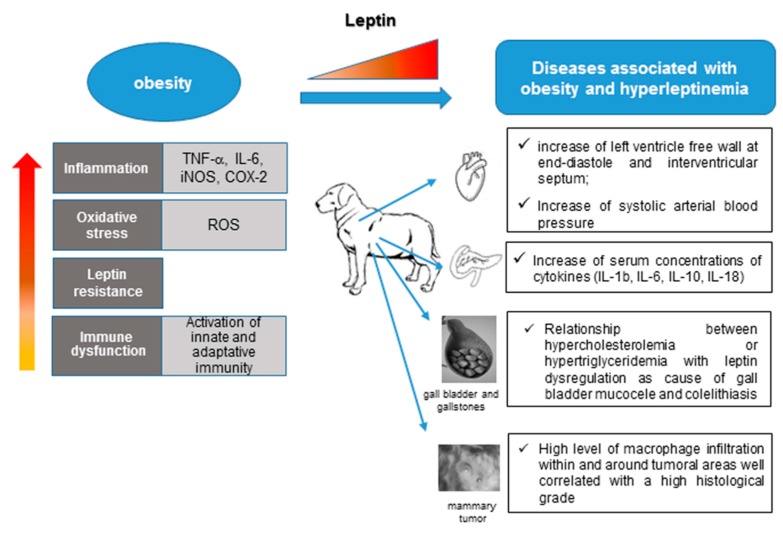
Obesity in dogs and diseases associated with hyperleptinemia.

Canine obesity has been associated with cardiac dysfunction [142,169,170,171]. Leptin has been observed in canine cardiovascular disease [172,173] (Figure 3). Varied morphologies of human obesity-related cardiac structural changes have been described and many include symmetric or asymmetric left ventricle hypertrophy (LVH) with or without left ventricular chamber dilatation [174,175]. In canine model, Adolphe et al. [170] described alterations in glucose, adipokines (leptin and adiponectin) and heart during obesity (Figure 3). Weight loss reversed these alterations. Piantedosi et al. [142] suggested the presence of myocardial concentric hypertrophy in obese dogs.

Systolic arterial blood pressure appeared to be higher in obese than in normal weight dogs (Figure 3). Similar cardiovascular findings and increased systolic blood pressure have been reported by Mehlman et al. [169]. In contrast, hypertension has not been related to canine obesity [176].

Obese dogs express alterations in cardiac function, insulin resistance, dyslipidaemia, hypo-adiponectinaemia and increased concentrations of inflammatory markers and leptin [171]. However, only few studies investigated the role of leptin in canine cardiac diseases [169,177,178]. In the heart, cardiomyocytes and endothelial cells produce leptin and express its receptor. In addition to changes in blood concentrations, functional auto- and paracrine effects may occur [179,180,181,182]. Leptin regulates the baseline physiology of the heart including myocyte contractility, hypertrophy, apoptosis, and metabolism [181,183,184]. Localized depots of epicardial or perivascular fat might also play physiological or pathological roles [183,185,186]. In cardiac disease (CDi) and in congestive heart failure (CHF), leptin significantly increased, suggesting that an increased metabolic rate is associated with high concentrations of catecholamines and proinflammatory cytokines present in CHF [187,188]. Furthermore, because of elicited central sympathoexcitatory effects, leptin participates in the neuro-humoral activation in heart failure [189]. Increased leptin has been associated with increased oxygen consumption and intracellular calcium release and decreased cardiac efficiency in vivo [179,186,190]. In CDi, leptin is involved in cardiac remodelling, characterized by cardiomyocyte hypertrophy and disruption of the extracellular matrix resulting in increased collagen deposition [185,186,191], which might contribute to cardiac dysfunction. Such a hormone protects cardiomyocytes from apoptosis, which plays an important role in the development of CHF [192]. Leptin might decrease cardiac hypertrophy, apoptosis, and inflammation in deficient leptin receptor mice [193]. Therefore, leptin can impact cardiovascular function by direct heart effects or by central nervous system responses and may represent a predictor of cardiovascular morbidity [179,185]. However, the role of leptin in development and progression of canine CDi and CHF is still poorly understood [172,173].

The role of leptin as a pathophysiological modulator has been described in other canine pathological conditions [194,195], besides cardiovascular diseases. Adipokines, especially resistin and visfatin, have been implicated in the development of acute pancreatitis (AP) in humans [196,197,198,199] and in experimental animal models [200,201,202]. However, little information is available about the circulating adipokine concentrations during the pathogenesis of AP in dogs. Recently, Paek et al. [203] described that leptin, resistin, and visfatin were significantly higher in the dogs with AP than in healthy dogs, whereas adiponectin concentration was significantly lower in AP than in healthy dogs. IL-1b, IL-6, IL-10, and IL-18 also increased in AP dogs [203]. These results suggest a potential role for adipokines in the development and modulation of AP in dogs (Figure 3).

In addition, leptin and its receptor play several physiological roles in the canine gallbladder (Figure 3). Gallbladder is not only a source of leptin, but it is also affected by autocrine/paracrine mechanisms [204]. Lee et al. [205] revealed an increased expression of leptin and leptin receptors in dogs with gallbladder mucocele (GBM), suggesting that such a hormone plays a role as a causative factor in GBM.

The relationship between serum triglyceride/cholesterol and leptin is still controversial [206,207]. Leptin may correlate with serum lipids in dogs [208,209]. A positive association between human hyperlipidaemia and gallstones has been described [210,211]. Recently, Lee et al. [212] described an increase in serum leptin during hyperlipidaemia and cholelithiasis occurrence in dogs.

Finally, leptin is known for its involvement in the regulation of reproductive functions. Such a hormone is important for uterine receptivity, implantation, placental growth, and maternal energy homeostasis in several species [213,214]. The uterus and placenta are also sources of leptin and targets of its actions during gestation in canine species. Leptin and leptin receptors are expressed both in the foetal and maternal sides of the placenta, thus, a role in placental physiology seems likely. The leptin signalling system may be one of the pathways involved in the establishment and maintenance of pregnancy and may also play a regulatory role in parturition in the bitch [215].

## 5. Conclusions

Leptin constitutes a relevant hormonal “actor” in obesity, immune-system homeostasis and in several associated metabolic-related as well as immune-mediated diseases [8]. Recent clinical studies on autoimmune disease patients demonstrated that high serum leptin levels may play a causal role in the disease progression and could represent a diagnostic marker for clinical application. It remains to be established if leptin could be a potential therapeutic target in treating human autoimmune diseases [30,216].

Circulating leptin correlates with fat mass and is considered a useful marker of adiposity in veterinary settings. However, no studies are available concerning other clinical applications of such a hormone, and about the involvement of leptin in canine immune-mediated diseases.

The growing worldwide scientific attention for obesity and leptin—in consideration of the important implications for quality of life in humans and animals—must motivate further studies, able to generate information on the molecular mechanism exerted by leptin in the course of the disease, and to therefore identify possible therapeutic targets for obesity as well as other associated diseases. Furthermore, the most recent interest in the study of obesity and related diseases in animal species appears to be intriguingly “translational” to better understand the human etiopathology of the metabolic syndrome. In fact, domestic animals, particularly the dog, represent the natural biological indicators of the habits of life in, correlating with human aspects. Therefore, the need for studies to understand the pro-inflammatory role of leptin and weight gain in canine diseases seems to be of great importance, not only for veterinary medicine, but also to protect human health and to contain health-related expenditure generated by many widespread chronic metabolic diseases.

## Abbreviations

apoE	Apolipoprotein E
AP	Acute pancreatitis
BCS	Body condition score
CDi	Cardiac disease
CHF	Congestive heart failure
CNS	Central nervous system
CRP	C-reactive protein
DC	Dendritic cells
GBM	Gallbladder mucocele
HF	Heart failure
IFN	Interferon
IL	Interleukin
JAK	Janus kinase
*LEPR*	Leptin receptor
LVH	Left ventricle hypertrophy
MAPK	Mitogen-activated protein kinases
MC	Mammary carcinoma
MCP-1	Monocyte chemoattractant protein-1
MS	Metabolic Syndrome
VEGF	Vascular growth factor
VL	Visceral Leishmaniasis
ROR	Retinoic acid-related orphan receptor
ROS	Reactive oxygen species
NK	Natural Killer
STAT	Signal transducer and activator of transcription
TNF	Tumour necrosis factor
Treg cells	Regulatory T cells
VSMCs	Vascular smooth muscle cells
